# Adiponectin Agonist ADP355 Attenuates CCl_4_-Induced Liver Fibrosis in Mice

**DOI:** 10.1371/journal.pone.0110405

**Published:** 2014-10-13

**Authors:** Pradeep Kumar, Tekla Smith, Khalidur Rahman, Natalie E. Thorn, Frank A. Anania

**Affiliations:** Emory University School of Medicine, Department of Medicine, Division of Digestive Diseases, Atlanta, GA, United States of America; The University of Hong Kong, Hong Kong

## Abstract

Liver fibrosis is a growing global health problem characterized by excess deposition of fibrillar collagen, and activation of hepatic stellate cells (HSCs). Adiponectin is known to possess anti-fibrotic properties; however a high physiological concentration and multiple forms circulating in blood prohibit clinical use. Recently, an adiponectin-like small synthetic peptide agonist (ADP355: H-DAsn-Ile-Pro-Nva-Leu-Tyr-DSer-Phe-Ala-DSer-NH2) was synthesized for the treatment of murine breast cancer. The present study was designed to evaluate the efficacy of ADP355 as an anti-fibrotic agent in the *in vivo* carbon tetrachloride (CCl_4_)-induced liver fibrosis model. Liver fibrosis was induced in eight-week old male C57BL/6J mice by CCl_4_-gavage every other day for four weeks before injection of a nanoparticle-conjugated with ADP355 (nano-ADP355). Control gold nanoparticles and nano-ADP355 were administered by intraperitoneal injection for two weeks along with CCl_4_-gavage. All mice were sacrificed after 6 weeks, and serum and liver tissue were collected for biochemical, histopathologic and molecular analyses. Biochemical studies suggested ADP355 treatment attenuates liver fibrosis, determined by reduction of serum aspartate aminotransferase (AST), alanine aminotransferase ALT) and hydroxyproline. Histopathology revealed chronic CCl_4_-treatment results in significant fibrosis, while ADP355 treatment induced significantly reversed fibrosis. Key markers for fibrogenesis–α-smooth muscle actin (α-SMA), transforming growth factor-beta1 (TGF-β1), connective tissue growth factor (CTGF), and the tissue inhibitor of metalloproteinase I (TIMP1) were also markedly attenuated. Conversely, liver lysates from ADP355 treated mice increased phosphorylation of both endothelial nitric oxide synthase (eNOS) and AMPK while AKT phosphorylation was diminished. These findings suggest ADP355 is a potent anti-fibrotic agent that can be an effective intervention against liver fibrosis.

## Introduction

Hepatic fibrosis is a reversible wound-healing response characterized by excess accumulation of extracellular matrix (ECM), mainly fibrillar collagens [1], [2]. Primary drivers of chronic liver injury that lead to fibrosis are viral infection, alcohol abuse, and non-alcoholic hepatic steatohepatitis (NASH) [1], [3], [4]. With recent reports of improvement in treatment of viral hepatitis, anti-fibrotic strategies in patients with NASH-related fibrosis and cirrhosis are urgently needed. Progression of liver fibrosis eventually leads to cirrhosis, which can be associated with hepatocellular carcinoma (HCC) and liver failure [5]. According to recent reports, HCC is the fifth most common cancer worldwide and the third leading cause of cancer-related death [6], [7]. Taken all together, assessment of effective anti-fibrotic agents that inhibit development of liver fibrosis could be useful in improving the prognosis of patients with chronic liver disease.

Activation of hepatic stellate cells (HSCs) plays a key role in the development of liver fibrosis since activated HSCs are the major contributors to dense ECM deposition when chronic liver injury is sustained [1]. In response to liver injury, vitamin A–storing HSCs undergo an activation process that results in transformation into hepatic myofibroblast–like cells that secrete chemokines, cytokines, type I fibrillar collagen, and the tissue inhibitor of metalloproteinase I (TIMP1)–a key molecule associated with inhibiting HSC apoptosis [8]–[10].

Though several peptides and natural products possess anti-fibrotic properties *in vitro* and *in vivo,* none have yet been used or approved in clinical studies by the US Food and Drug Administration to treat liver fibrosis. Adiponectin is a 30 kDa protein adipocytokine synthesized and secreted by white adipose tissue. A primary function of adiponectin is to reduce systemic insulin resistance by activation of AMPK [11]–[13]. Adiponectin relatively circulates high concentrations in blood at 3–30 µg per ml in trimeric, hexameric, and multimeric forms [14]–[17]. Adiponectin signals its biological effects primarily by binding two distinct transmembrane receptors, adiponectin receptors 1and 2, which are down-regulated as is adiponectin in obesity, obesity-linked insulin resistance, and type 2 diabetes mellitus [18]–[21]. Adiponectin-receptor binding activates a canonical intracellular signaling pathway by activation of a fuel-sensing cellular enzyme, 5′ adenosine monophosphate-activated protein kinase (AMPK). We and others have also identified an anti-fibrotic role for adiponectin, which can serve as a plausible cytokine offering protection against leptin and carbon tetrachloride (CCl_4_)-mediated hepatic fibrogenesis [22]–[25]. However, based on the adiponectin amino acid sequence, Otvus et al. designed, synthesized, and characterized a peptide (ADP355) that mimics key biological functions of adiponectin *in vitro* as well as *in vivo* [26].

There is significant interest in using targeted nanoparticles to deliver drugs, or bioactive compounds, to cells *in vivo* and *in vitro* [27], [28]. Gold nanoparticles comprise a novel class of nanoparticles that have significant potential for drug delivery [29]. Using gold nanoparticle can have many advantages for drug delivery, such as higher bioavailability, efficacy, and preferable accumulation in the liver for detoxification if the nanoparticles are larger than 8 nm [30], [31]. To date, there are a very limited number of studies focused on the potential effects of nanotherapeutics in chronic liver diseases. We generated gold nanorods coupled and stabilized with ADP355 to evaluate the effect of ADP355 in liver fibrosis using eight week old mice (C57BL/6J) that were gavaged with CCl_4_ for six weeks. We report that gold nanoparticle delivery of ADP355 resulted in significant reduction of liver fibrosis and revealed key changes associated with myofibroblast function.

## Materials and Methods

### Synthesis of ADP355 and nanoparticle conjugation

ADP355 (DAsn-Ile-Pro-Nva-Leu-Tyr-DSer-Phe-Ala-Dser-NH2) was synthesized by American Peptide Company (Sunnyvale, CA) [26]. We synthesized a 10 nm *in vivo* injectable gold nanoparticle-ADP355 conjugate (Nanopartz Inc., Loveland CO).

### Animal models and induction of hepatic fibrosis

Eight-week-old male C57BL/6J mice were used for this study (Jackson Laboratory, Bar Harbor, Maine; Stock no.000664). All procedures were performed in accordance with the Guide for Care and Use of Laboratory Animals of the National Institute of Health. The protocol was approved by the Institutional Animal Care and Use Committee of (IACUC) of Emory University (Permit No. DAR2001591-040415BN). Animals were euthanized under isofuran anesthesia. All animals were housed in a temperature-controlled environment with 12-h dark and light cycles. The mice were divided into the following four cohorts: (1) mice which received olive oil every other day (2 ml/kg body weight) for 6 weeks and served as the control group (Saline); (2) mice that received CCl_4_-gavage every other day for 6 weeks by gavage (2 ml/kg body weight; CCl_4_ diluted 1∶1 in olive oil); (3) mice that received CCl_4_-gavage and gold nanoparticle intraperitoneally every other day for two weeks after four weeks of CCl_4_-gavage; and (4) mice that received CCl_4_-gavage plus gold nanoparticle-ADP355 [0.5 mg/kg body weight]: intraperitoneally every other day for two weeks-after four weeks of CCl_4_-gavage). The concentration of ADP355 we used in the present study was based on a prior publication [26]. We determined using UV-VIS assay the localization of the nanoparticles 2 h following injection to confirm that the majority of particles were present in liver tissue. We also confirmed the concentration of the gold-nanoparticle-ADP355 by performing spectroscopy to identify the compound particle as spectroscopically identified by the manufacturer.

### Sirius red staining

Liver sections were fixed in 10% neutral buffered formalin for 24 h and transferred to 70% ethanol before imbedding in paraffin blocks. Paraffin embedded liver tissues were cut into 5–µm thick sections. Deparaffinized sections were incubated for 60 min with Pico-Sirius red solution (Abcam, Cambridge, MA) followed by a brief rinse with acetic acid (0.05%). Sections were dehydrated by washing with absolute alcohol [32]. Sections were observed with a light microscope (Axioplan2; Carl Zeiss, Hallbergmoos, Germany).

### Hematoxylin and Eosin staining

Five–micron paraffin-embedded liver sections were deparaffinized and washed with water. Hydrated tissue sections were incubated with Mayer’s hematoxylin for 5 min followed by vigorous washing in running tap water. Sections were counter–stained with 1% eosin for 2 min. Eosin stained sections were washed with water and dehydrated with alcohol. Dehydrated sections were washed with xylene. Images were taken with a microscope (Axioplan2; Carl Zeiss, Hallbergmoos, Germany).

### Serum alanine amino aminotransferase and aspartate aminotransferase assay

Serum ALT and AST levels were determined as per manufacturer instructions (Sigma-Aldrich St. Louis, MO).

### Hydroxyproline assay

Hydroxyproline assay to quantify collagen content were performed using a colorimetric method described by the manufacturer (Bio Vision, Milpitas, CA), In brief, 10 mg of liver tissue were excised and homogenized in 100 µl of sterile MQ water followed by hydrolysis in 12N HCl (100 µl) at 120°C. After 3 h, 5 µl of tissue lysate was transferred to a 96-well plate and incubated at 37°C for 16 h to evaporate the acid. Samples were incubated with equal amounts of chloramine T and Ehrlich’s reagents for 30 min at 65°C. Absorbance was recorded at 560 nm with an ELISA plate reader (Synergy BioTek, Winooski, VT).

### Immunohistochemical staining of α-SMA

A formalin–fixed and paraffin-embedded liver section (5 µm) was subjected to antigen retrieval by heating in a microwave oven for 10 min in citrate buffer (pH 6.0). Sections were blocked for 1 h with 5% BSA in TBST followed by incubation with monoclonal anti- α-smooth muscle actin (α-SMA) primary antibody (dilution 1∶50; Pierce, Rockford, IL) at 4°C overnight. Immuno-staining was performed using a DAB substrate kit according to manufacturer instructions (Abcam, Cambridge, MA). Sections were counter–stained with Mayer’s hematoxylin. Image was scan by using Leica Microsystem scanner (Leica Microsystem Inc. Buffalo Grove, IL).

### RNA extraction and qRT-PCR analysis

Total RNA was extracted from snap-frozen liver tissues (20–30 mg) using the RNeasy Mini Kit according to manufacturer’s instructions (Qiagen, Valencia, CA). Briefly, liver tissues were washed with RNA Later and then homogenized in RLT buffer. To avoid genomic DNA contamination, the RNeasy mini column was treated with DNAse I for 15 min at room temperature (Qiagen, Valencia, CA). For the first-strand cDNA synthesis, 1 µg of total RNA was extended to 20 µl total reaction volume using the Bio-Rad’s iScript cDNA synthesis kit, according to manufacturer (Bio-Rad, Hercules, CA) instructions. The following primers were used in this study: α-SMA (forward) (5′-CCGAGATCTCACCGAC-3′); (reverse) (5′-TCCAGAGCTACATGACACAG-3′); TIMP1 (forward) (5′-CACGGGCCGCCTAAGGAACG-3′); (reverse) (5′-GGTCATCGGGCCCCAAGGGA-3′); MMP13 (forward) (5′-GCCCTGGGAAGGAGAGACTCCAGG-3′); (reverse) (5′- GGATTCCCGCAAGAGTCGCAGG-3′); desmin (forward) (5′-AGCGTGACAACCTGATAGACG-3′); (R) (5′-TGAAGCTCACGGATCTCCTCT-3′); TGFβ1 (forward) (5′-GGGAGACCCCACCCCACAAG-3′); (reverse) (5′- ACAAAGCGAGCACCGCCTCG-3′); 18 s (forward) (5′-GCAATTATTCCCCATGAACG-3′); (reverse) (5′-GGCCTCACTAAACCATCCAA-3′). Quantitative reverse-transcriptase polymerase chain reactions (qRT-PCR) were performed using the IQ™ SYBR Green Supermix (Bio-Rad, Hercules, CA) according to standard protocol. The cycle conditions were 95°C for 15 s, 55°C for 15 s, and 72°C for 20 s, for 40 cycles. The qRT-PCR assays were performed in triplicate using the Mastercycler–Eprealplex (Eppendorf, Hamburg, Germany) with internal controls for 18 s mRNA. The amplified PCR products were quantified by calculating the cycle thresholds (CTs) for the individual target gene and 18 s mRNA. The comparative 2^−ΔΔCT^ method was adopted for calculation of fold changes relative to the control [22].

### SDS-PAGE and Western blot analysis

Snap-frozen liver tissues (60–80 mg) were homogenized and sonicated in 300 µl RIPA lysis buffer (Sigma-Aldrich, St. Louis, MO) containing a protease inhibitor cocktail and phosphostop (Roche Diagnostics, Indianapolis, IN). Total extracts were collected by centrifugation at 14000 g for 10 min at 4°C. Total protein concentration was determined using a BCA protein assay kit (Pierce, Rockford, IL). Then 20–50 µg total protein was resolved on 4–12% precast SDS-PAGE gels (Invitrogen, Grand Island, NY). Proteins were transferred onto a polyvinylidene fluoride membrane (Bio-Rad). Membranes were blocked to minimize non-specific binding in TBST containing 5% nonfat dry milk for 1 h at room temperature. The membrane was incubated overnight with primary antibody (1∶500 dilution in TBST and 5% BSA) at 4°C. The primary antibodies were purchased from following manufacturer; CTGF, TGF-β1and MMP13 (Santa Cruz Biotechnology, Dallas, Texas); p-AMPKα tyr172, AMPKα, p-eNOS ser1177, eNOS, pAKT ser473 and AKT (Cell Signaling, Beverly, MA); anti-actin, α-SMA and anti-desmin (Sigma-Aldrich, St. Louis, MO); anti-mTIMP1 (R&D Systems, Minneapolis, MN). Membranes were then incubated with secondary antibody conjugated with horseradish peroxidase for 1 h followed by vigorous washing with TBST.

Protein bands were detected using a SuperSignal West Femto chemiluminescent HRP antibody detection reagent (Pierce, Rockford, IL) and visualized using Biospectrum BioChemi 500 Imaging System (UVP, Upland, CA). Densitometric analysis of resolved proteins was performed to quantify protein band intensity with VisionWorks Software, version 6.8 (UVP, Upland, CA).

### *In situ* Zymography

The hepatic collagen-degrading activity in liver tissue was analyzed using *in situ* positive fluorescent collagen I zymography with as described previously [33]. Briefly, DQ collagen (10 µg/ml), type I from bovine skin, fluorescein conjugate (Life technologies, Grand Island, NY) was dissolved in 50 mM Tris, pH 7.5, 150 mM NaCl. Cryosections (5 µM) were briefly rinse with PBS and 100 µl of DQ solution was applied. The slides were incubated at room temperature for 16 h. Fluorescent images were captured with a microscope (Axioplan2; Carl Zeiss, Hallbergmoos, Germany).

### Statistical analysis

Data represent the mean ± SEM. Statistical analysis was performed using the student *t* test, the Graph-Pad Prism software version 5.04 for Windows (GraphPad Software, La Jolla, CA). *p*<0.05 was considered statistically significant.

## Results

### ADP355 administration attenuates CCl_4_ induced liver injury and collagen degradation

**Figure 1:**
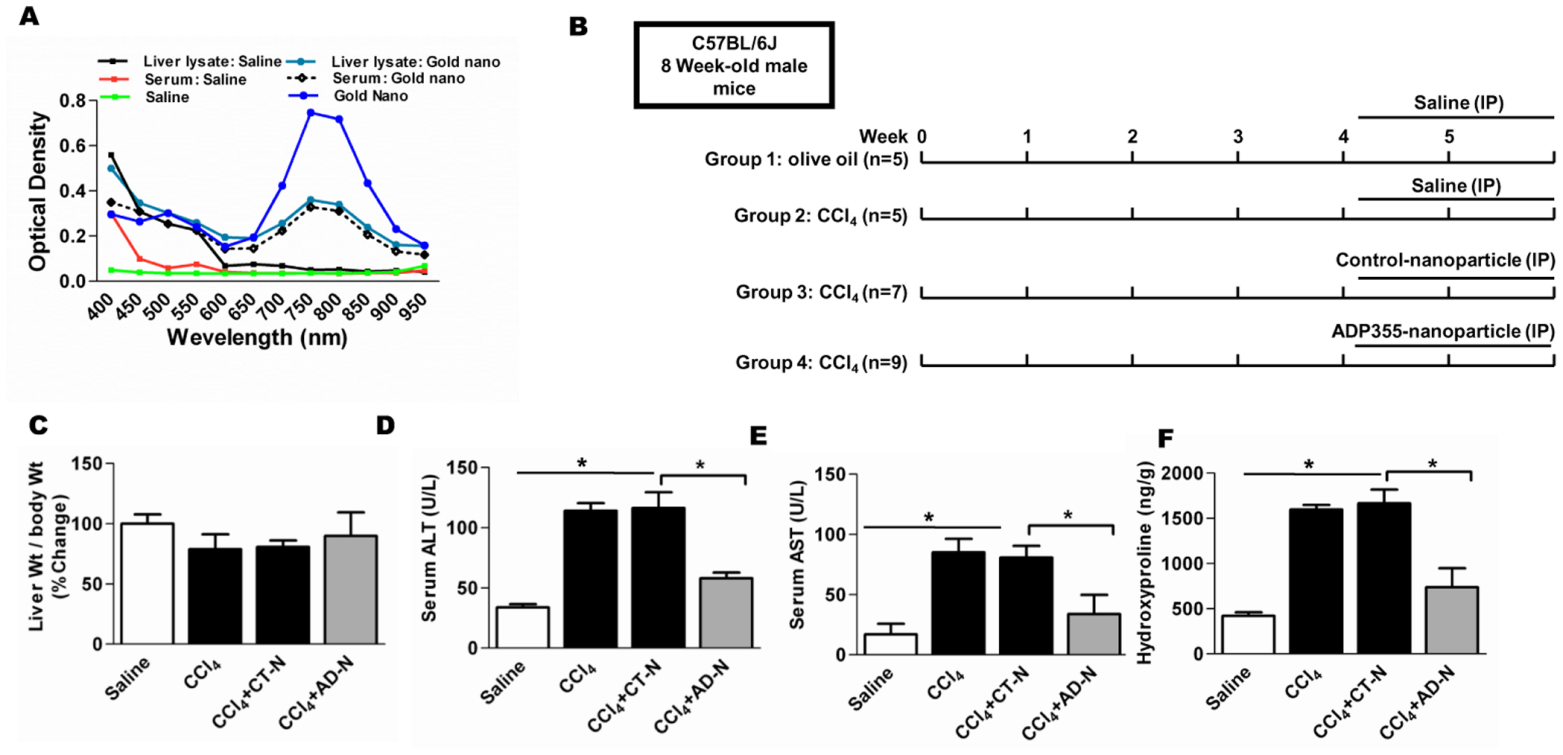
Experimental design and biochemical analysis. **A**) UV-VIS spectra of gold nanoparticle, 20 µl of ADP355-gold nanoparticle conjugate were injected intraperitoneally into mice. Following 2 h treatment, mice were euthanized, and serum samples and homogenized liver tissues were collected for UV-VIS spectra. Control mice received an equal volume of saline per injection. Saline and ADP355-gold nanoparticles were also included for analysis. **B**) Experimental design of the schedule for CCl_4_ and nanoparticle injections. **C**) Percent change in liver weight divided by body weight of mice. **D**–**E**) Serum ALT and AST levels markers of chronic liver injury. Goldn-ADP355 showed significant reduction in CCl_4_-induced serum ALT and AST. **F**) Liver hydroxyproline was determined by acid hydrolysis; hydroxyproline content was significantly increased in CCl_4_-gavaged mice compared to normal control mice, while hydroxyproline content was significantly reduced in CCl_4_–ADN treated mice (*p<0.05) (N = 5 mice/cohort).

In order to first investigate targeted nanoparticle delivery to the liver and circulation, mice were injected with gold nanoparticles intraperitoneally and we assessed serum and liver lysates for gold nanoparticle bio-distribution by using UV-VIS spectra, Fig. 1A. The presence of gold nanoparticles within the liver and white adipose tissue (WAT) was further confirmed by dark field microscopy as shown in figure S1. The experimental design of the CCl_4_-gavage and nanoparticle treatment is presented in Fig. 1B. After the experiment was completed, and mice were euthanized, we examined the change in the ratio of liver weight to body weight and compared the ratio among the different treatment groups though we did not see significant change in the ratio of liver weight compare to control (Fig. 1C). As shown in Fig. 1D–E, CCl_4_ and CCl_4_–control nanoparticle (CCl_4_ or CCl_4_–CTN)–treatment significantly increased serum ALT and AST compared to controls, whereas the ALT and AST levels were significantly reduced in CCl_4_-AD-nanoparticle (CCl_4_–ADN) treated mice.

Hydroxyproline, a non-proteinogenic amino acid predominantly formed by post-translational hydroxylation of protein found in collagens is used to approximate liver tissue collagen concentration and to determine the effectiveness of an anti-fibrotic effect as evidence by a reduction in serum hydroxyproline. As shown in Fig. 1F, hydroxyproline content was significantly higher in CCl_4_-gavaged and CCl_4_-CTN treated mice than in the saline injected mice. Following CCl_4_-ADN injection, hepatic hydroxyproline contents were significantly reduced (Fig. 1E). Taken together these data suggest that gold nanoparticle delivery of ADP355 significantly attenuates CCl_4_-induced liver fibrosis in mice.

**Figure 2:**
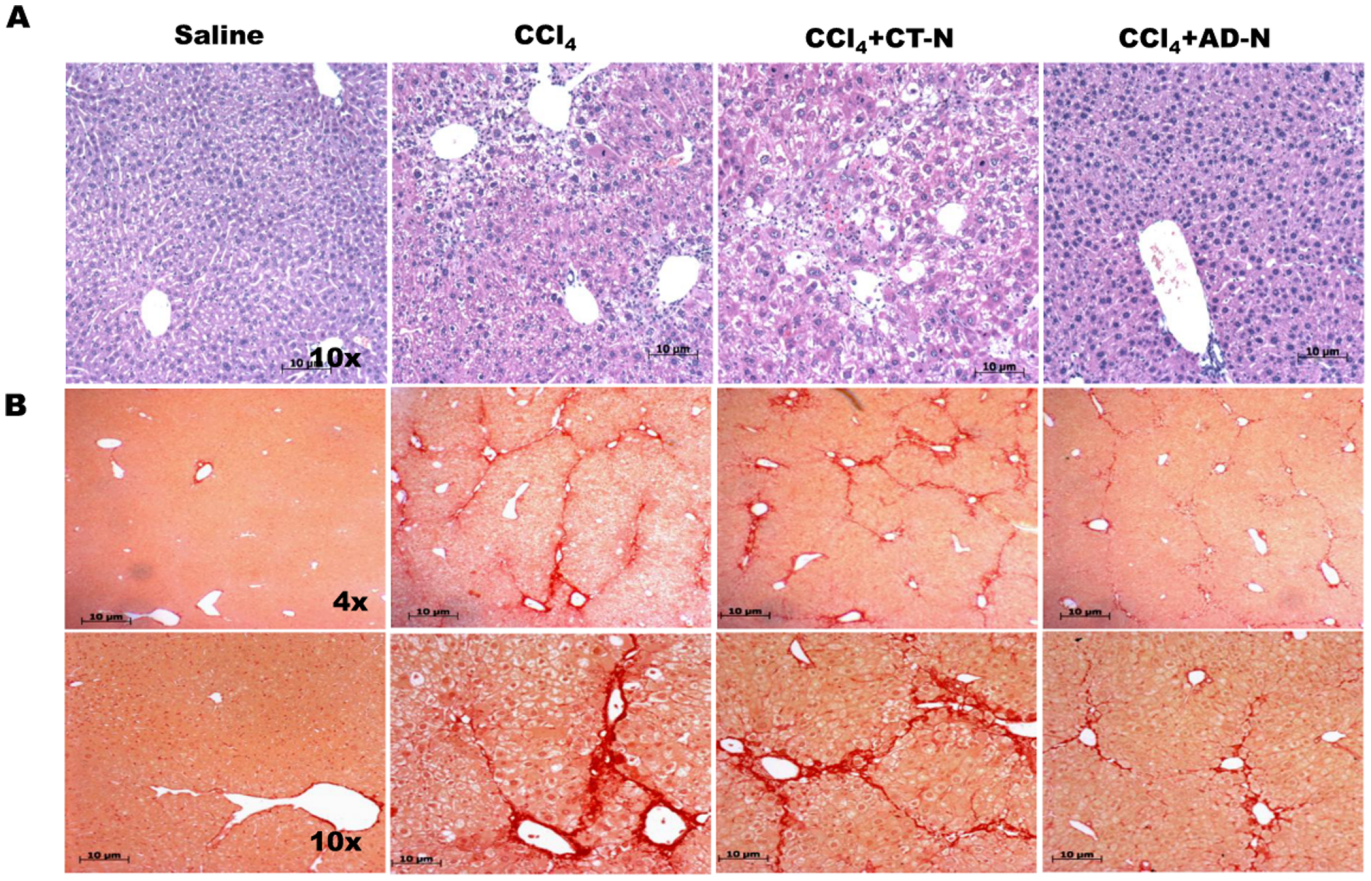
Histological analysis of CCl_4_-induced chronic liver injury. A) Representative H&E stained liver section are shown. B) Representative Sirius Red staining liver sections obtained from control (saline group) revealed normal lobular structure while in CCl_4_-gavaged mice liver sections reveal extensive collagen deposition and bridging fibrosis. The degree of collagen deposition was significantly decreased in CCl_4_–ADN treated mice (N = 5 mice/cohort).

The effect of ADP355 in mitigating CCl_4_-induced fibrogenesis was also corroborated with histopathologic analysis. Representative H&E staining of liver sections are presented in Fig. 2A. As shown in Fig. 2A, CCl_4_ treatment resulted in necrosis and fibrotic septa between parenchymal nodules. ADP355 administration for two weeks improved histological parameters with a significant reduction in micro- and macrovesicular steatosis (Fig. 2A). Sirius Red staining revealed CCl_4_ administration alone significantly increased collagen deposition in perisinusoidal areas; however, ADP355 administration for two weeks resulted in a remarkable reduction in the size of the stained area of fibrous dense tissue as shown in Fig. 2B–inspite of concomitant CCl_4_-gavage.

### ADP355 attenuates CCl_4_-induced α-SMA activation

**Figure 3:**
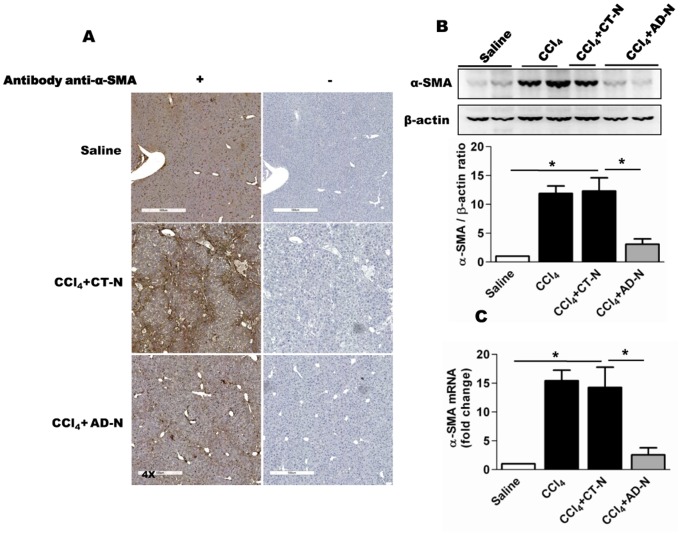
Effect of ADP355 on CCl_4_-induced liver fibrosis in mice. **A**) Representative immunohistochemical (α-SMA) stained liver sections obtained for various treatments outlined (left panel); antibody control were presented in right panel. The original magnification was 4X. **B**) Representative Western blot analysis and densitometry for α-SMA liver lysates obtained from saline, CCl_4_, CCl_4_–CTN and CCl_4_–ADN treated mice. **C**) Hepatic α-SMA mRNA expression was assessed by qRT-PCR compared to housekeeping gene 18 s. *p<0.05 (N = 5 mice/cohort).

Previous studies identified that elevated expression of alpha smooth muscle actin (α-SMA) is a marker of activated HSCs [34], [35]. As shown in Fig. 3A, α-SMA staining was markedly increased in CCl_4_-treated mice compared to the control groups, whereas it was markedly attenuated in liver sections obtained from ADP355 treated mice. Western blot and qRT-PCR analyses from liver tissue obtained from different arms were also performed. The IHC data demonstrated significant reduction in α-SMA mRNA (Fig. 3B) and protein expression in ADP355 treated mice compared to CCl_4_-gavaged mice (Fig. 3C). To analyze whether CCl_4_-induced α-SMA deactivation was a specific consequence of ADP355 treatments, we used alternative approaches. Mice were gavaged with CCl_4_ for three weeks followed by intraperitoneal injection of FLAG peptide (H-Asp-Tyr-Lys-Asp-Asp-Asp-Asp-Lys-OH) (1 mg/kg body weight), ADP355 (1 mg/kg), CTN and ADN (0.5 mg/kg) for two weeks along with CCl_4_. Western blot analysis shows, ADP355 and ADN treatments significantly attenuates the CCl_4_-induced expression of α-SMA despite the presence CCl_4_ while FLAG peptide treatment failed to alter the CCl_4_-induced α-SMA expression compared to CCl_4_ gavaged mice (Fig. S2).

**Figure 4:**
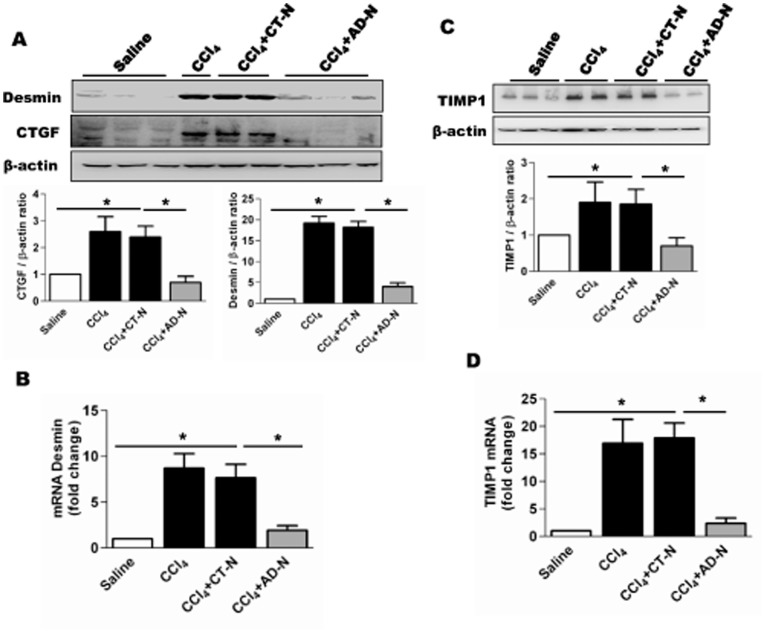
Figure 4. ADP355 attenuates CCl_4_-induced desmin, CTGF and TIMP1 expression in mice. **A**) Representative Western blots analysis of liver lysates: Desmin (Top panel); CTGF (middle); β-actin expression (lowest panel). Bar graph represents densitometric quantification compared to saline groups. **B**) mRNA expression of desmin from liver tissues. **C**) Western blot of TIMP1 in lysates obtained from liver tissues. **D**) Hepatic mRNA expression of TIMP1 (N = 5 mice/cohort).

Desmin, another well-documented marker of myofibroblasts was analyzed by qRT-PCR expression and Western blot analysis (Fig. 4A–B). We observed significant reduction of desmin expression in the CCl_4_-ADN group compared to CCl_4_ and CCl_4_-CTN treated groups, as shown in Fig. 4A–B. Expression of connective tissue growth factor (CTGF) is elevated in fibrotic liver and co-localizes with α-SMA positive HSCs [36], [37]. Western blot analyses of liver lysates from different groups (Fig. 4A) reveal ADP355 administration significantly attenuates CCl_4_-induced CTGF protein expression in whole liver lysates.

### ADP355 reduces TIMP1 expression

**Figure 5:**
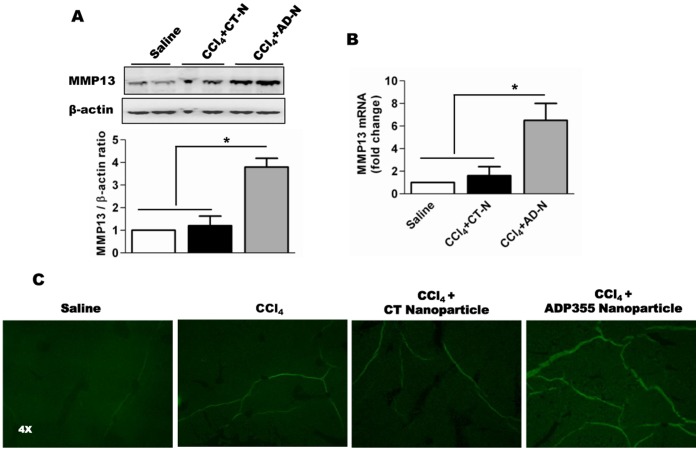
ADP355 induced MMP13 expression in CCl_4_-induced liver tissues of mice. **A**) Representative Western blot of MMP13 from liver lysates under four experimental conditions described previously. **B**) mRNA expression of MMP13 outlined in (**A**). **C**) *In situ* collagen zymography; collagen I-degrading activity in cryopreserved liver sections. Degraded collagen was detected by FITC–labeled DQ–collagen I. The original magnification was 4X (N = 5 mice/cohort).

Previous studies demonstrated that proteolytic activity of collagen is regulated by matrix metalloproteinase (MMPs), which are in turn regulated by their endogenous inhibitors, TIMPs [23]. During hepatic fibrogenesis, TIMP1 expression is markedly increased and has been shown to be a key player in preventing myofibroblast apoptosis. To determine the effect of ADP355 on expression of TIMP1, qRT-PCR and Western blot analyses were performed (Fig. 5C, D). Expression of the protein was significantly elevated in the CCl_4_-treated mice compared to controls (Fig. 4C). After ADP355 administration, TIMP1 expression was significantly attenuated compared to CCl_4_-gavaged mice, as assessed by Western blot and qRT-PCR analysis (Fig. 4D).

We examined the expression of MMP13, an analogue of human MMP1 and a key protease for type I collagen. Carbon tetrachloride induced the expression of MMP13, while ADP355 administration further induced expression of MMP13 (Fig. 5A, B). The increased expression of MMP13 may have resulted from reduced TIMP1 expression indicating that ADP355 can induce fibrillar collagen degradation in mice. We did assess collagen degradation in this report by performing *in situ* collagen zymography on cryosections of frozen liver tissues. In the control group, we found negligible collagenase activity while in CCl_4_ groups it was elevated; ADP355 administration significantly increased collagenase activity, as evident in Fig. 5C.

### ADP355 attenuates TGF-β1 expression

**Figure 6:**
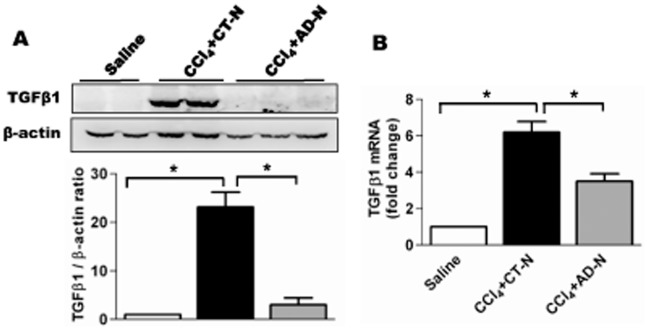
ADP355 reduced TGF-β1 expression. **A**) Representative of Western blot analysis for TGF-β1 (Top panel), densitometric analysis presented in bottom panel. **B**) mRNA expression of TGF-β1 total RNA obtained from whole liver tissue lysates, data are represented as the TGF-β1 expression relative to 18 s housekeeping gene, *p<0.05 (N = 5 mice/cohort).

Transforming growth factor, beta 1 (TGF-β1) is a major pro-fibrogenic cytokine associated with organ fibrogenesis [38]. We examined the effect of ADP355 on the expression of mRNA and protein levels by qRT-PCR and Western blot analysis. As shown in Fig. 6 A–B, mRNA and protein levels of TGF-β1 in liver tissues were significantly increased in the CCl_4_ group compared to saline-treated mice while ADP355 significantly reduced mRNA and protein expression of TGF-β1 compared to CCl_4_-gavaged mice treated with empty nanoparticle.

### ADP355 induced eNOS and AMPK phosphorylation

**Figure 7:**
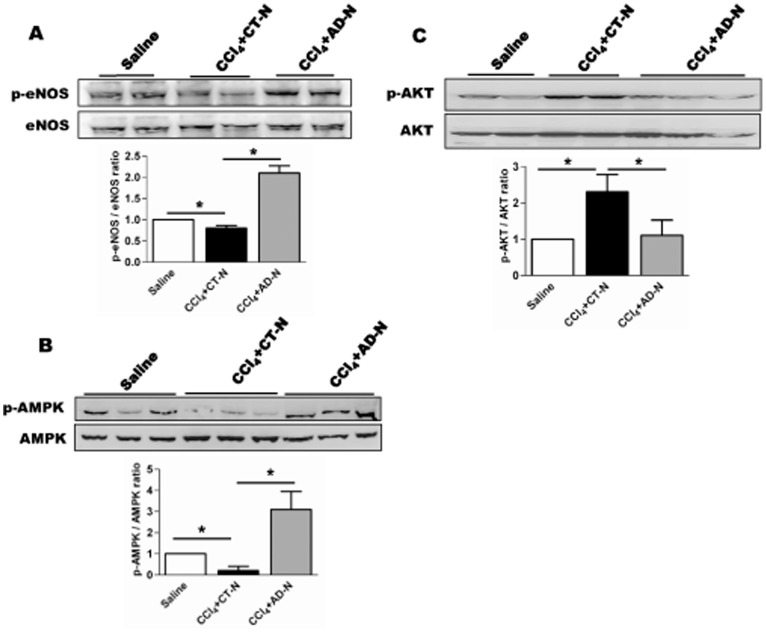
ADP355 modulates eNOS, AMPK and Akt phosphorylation *in vivo*. **A**–**B**) Western blot analysis showed that CCl_4_ reduced the phosphorylation of eNOS and AMPK, while ADP355 treatment significantly increased the phosphorylation of eNOS ser1177 and AMPK tyr172 residue. **C**) Western blot and densitometric ratio of p-AKT ser473/AKT, *p<0.05 (N = 5 mice/cohort).

Several *in vivo* and *in vitro* studies have shown that adiponectin induces the production of nitric-oxide (NO) as a consequence of increased endothelial nitric oxide-synthase (eNOS) activity, which is mediated by phosphorylation of AMPK [39]–[41]. To determine the activation of eNOS, numerous kinase and phosphorylation sites have been identified, but Ser1177 appears to be the most critical eNOS residue. Western blot data suggest that CCl_4_ treatment significantly diminished AMPK phosphorylation, or activation as well as eNOS phosphorylation compared to controls (Fig. 7A–B). Interestingly, ADP355 administration significantly induced phosphorylation of AMPK and eNOS (Fig. 7A–B). Finally, Western blot analyses also revealed the phosphorylation of Akt was significantly up-regulated in the CCl_4_-treated group compared with the control group, while ADP355 treatment significantly attenuates the CCl_4_-induced phosphorylation of Akt (Fig. 7C). Since Akt is important in cell survival, the Akt data would provide evidence as to why activated HSCs are normally resistant to apoptosis. We performed immunofluorescent microscopy of liver section stained with p-AKT ser473 from respective mouse cohorts to determine whether p-AKT staining co-localized with α-SMA positive cells. Surprisingly, we observed intense staining of p-AKT and α-SMA in CCl_4_-gavaged mice; furthermore, p-AKT staining co-localized with α-SMA positive cells (Fig. S3).

## Discussion

The present study demonstrates that a small synthetic peptide analogue of adiponectin, ADP355, can reverse CCl_4_-induced hepatic injury and fibrogenesis through multiple anti-fibrogenic mechanisms. We found that ADP355 significantly reduced expression of α-SMA, desmin, CTGF, TGF-β1, and TIMP1, while ADP355 up-regulated the expression of MMP13. The mechanism for this protective effect could be a result that ADP355 effectively reverses the activation of HSC *in vivo*, or at least shifts HSC phenotype into reversion as Brenner and colleagues have recently shown in lineage tracing experiments [42].

Circulating adiponectin occurs as trimeric, hexameric, or oligomeric complexes of monomers and cleavage to produce the C-terminal globular domain, and this event has also been proposed as an important regulatory step in adiponectin action since this C-terminal fragment can mediate potent physiological effects. Indeed, the C-terminal half of the 149–166 residue-domain (active site) is located inside the trimeric form, but our peptide fragment, represented by peptide 25 and ADP355, is actually at the other end of the protein and can easily interact with the receptor. In fact, a potential mutation site that inhibits trimer formation and secretion of adiponectin from cells was mapped to the C-terminal end of peptide 26, outside the 25 and ADP355 peptide boundaries. Having said this, it is not clear if the trimer or multimeric forms interact with the receptor. It is very likely, therefore, all residues, inside or outside the trimeric bundle, will be exposed upon proteolytic processing and can interact with AdipoR. Finally, human recombinant globular adiponectin produced in *E. coli* is a homodimeric, non-glycosylated polypeptide that can produce AdipoR-mediated biological effects in different cell types. Hence, in discussion with our colleagues who have used ADP355 in other biological studies the effects of ADP355 are related to native function of naturally occurring adiponectin in mammals.

Carbon tetrachloride-induced liver fibrosis has been extensively studied as a *in vivo* fibrosis model [43]. Carbon tetrachloride is metabolized in the liver by cytochrome P450, forming the free radical CCl_3_ which results in hepatocyte necrosis that promotes an inflammatory response in the injured liver [44]. Hepatocyte damage is the initial trigger for hepatic fibrogenesis, and serum ALT and AST are the most commonly used biochemical markers. In this study, mice were gavaged with CCl_4_ for four weeks to establish liver fibrosis before introduction of ADP355. Histological analysis showed CCl_4_ treatment resulted in necrosis and significant collagen accumulation with development of nodules and septa. By using a small synthetic ten amino acid sequence we convincingly demonstrate that administration of ADP355 for two weeks significantly improved hepatic histology and significantly suppressed and eliminated hepatic fibrosis. These findings were demonstrated histologically whereby thickness of bridging between fibrotic septa was diminished, as well as by biochemical assays which showed serum heightened serum ALT and AST activities markedly increased in CCl_4_-gavaged mice, were profoundly reduced by ADP355.

There is enormous interest in modifying existing drug delivery in improving pharmacokinetics, reducing non-specific side-effects, and permitting higher dose delivery to targeted organs of interest. In recent years, interest in the use of gold nanoparticles to enhance drug delivery was directed. It has been suggested that gold nanoparticles 1–2 nm in diameter are normally toxic whereas larger gold nanoparticles are nontoxic [45]. However, a recent study demonstrated that treatment of gold nanoparticle–induced hepatic macrophages activation which significantly exacerbates liver damage and immune-mediated hepatitis in mice [29]. In the present study, we did not observe any significant liver toxicity however, further studies will be required. Our findings may be the due to the size of the nanoparticle we created, as well as the overall aggregate size of the ADP355-gold nanoparticle conjugate. Nonetheless, we demonstrated that the nanoparticle was effectively delivered to the liver (Fig. 1A) using UV-VIS. Consistent with previous finding we also observed nanoparticle distribution in white adipose tissue [46].

In chronic liver disease, HSCs, the major source of ECM in the liver, undergo a process called trans-differentiation from a resting, vitamin–A storing phenotype to a myofibroblast-like phenotype characterized by expression of α-SMA [1]. In liver tissue, this is an important indicator of HSC activation. Inhibition or reduction of α-SMA may be an important marker for reversal of liver fibrosis, and would be expected to be lower expression of α-SMA in HSCs. Our results shown here indicate that the expression of α-SMA in mouse liver tissues significantly increased after CCl_4_ administration whereas gold-nano-ADP355 treatment significantly reduced α-SMA expression at protein and mRNA levels.

Suppression of TGF-β1 expression can also ameliorate liver fibrosis [38]. This study reveals that mRNA and protein expression of hepatic TGF-β1 in CCl_4_-induced mice were significantly higher compared to controls, but significantly lower in ADP355 treated mice. The CTGF also plays a key role in progression of liver fibrosis in mice. Small-interfering RNA–mediated knock-down of CTGF can prevent activation of rat HSCs and reduce ECM production [47]. Therefore, inhibition of CTGF expression is an important target for development of anti-fibrotic therapy. Based on these observations and concepts, we determined the effect of ADP355 on expression of CCl_4_–stimulated CTGF in liver tissues. Western blot analysis clearly showed that CCl_4_ increased CTGF expression and that ADP355 significantly attenuated CTGF expression in the mouse liver.

Recent studies show collagen and other extracellular matrix proteins are degraded by MMPs and that expression of MMPs is regulated by their endogenous inhibitor, TIMP1 [48], [49]. We have shown adiponectin regulate the expression and activity of MMPs and TIMPs in HSCs. In this study, expression of MMP13 in mouse liver was induced in CCl_4_-gavaged mice while nanoparticle-ADP355 conjugates resulted in significantly increased expression of MMP13. While we did not measure either TIMP1 or MMP13 activity here, we did perform a zymography to measure the effect of ADP355 on collagenase activity. This treatment revealed a stunning increase in such activity compared to control activity.

There are numerous reports demonstrating that adiponectin increases NO production by activating the AMPK–eNOS signaling pathway [50]–[52]. Our data suggests that CCl_4_ reduced the phosphorylation of AMPK and eNOS while administration of ADP355 induced the phosphorylation of AMPK and eNOS in lysates obtained from liver mouse liver tissues.

In summary, the results of this study confirm that the adiponectin analogue ADP355 attenuates CCl_4_-induced hepatic fibrosis in mice. Our study also suggests that ADP355 induces expression of MMP13, AMPK and eNOS phosphorylation in the liver. These data suggest that ADP355 might be a potential anti-fibrogenic candidate for the treatment of hepatic fibrosis.

## Supporting Information

Figure S1 **Gold nanoparticle distribution in liver and white adipose tissue.** Dark field microscopic images of liver (Top panel) and white adipose tissue (bottom panel) in saline injected mouse (left panel) and ADP355-N injected mouse (right panel) (original magnification 10X). Arrowhead showing the distribution of gold nanoparticles in ADN injected mouse.
(TIF)

Figure S2 **ADP355 attenuates CCl4-induced α-SMA expression.** Representative Western blot for α-SMA liver lysates obtained from different cohorts (N = 3 mice/cohort).
(TIF)

Figure S3 **p-AKT co-localized with α-SMA positive cells:** Representative images of immunofluorescent staining reveal both p-AKT and α-SMA co-localized in liver sections obtained from CCl_4_ gavaged mice.
(TIF)
