# Supplementary figures and images for: Adiponectin Agonist ADP355 Attenuates CCl4-Induced Liver Fibrosis in Mice
Source: PLoS One. 2014 Oct 13;9(10):e110405. doi: 10.1371/journal.pone.0110405 (PMC4195748; doi:10.1371/journal.pone.0110405)

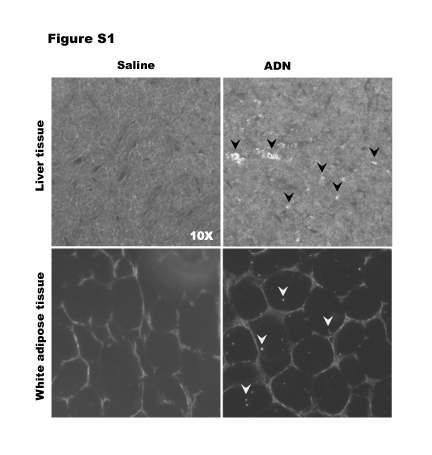

Supplement: Figure S1 — Gold nanoparticle distribution in liver and white adipose tissue. Dark field microscopic images of liver (Top panel) and white adipose tissue (bottom panel) in saline injected mouse (left panel) and ADP355-N injected mouse (right panel) (original magnification 10X). Arrowhead showing the distribution of gold nanoparticles in ADN injected mouse. (TIF) [file pone.0110405.s001.tif]

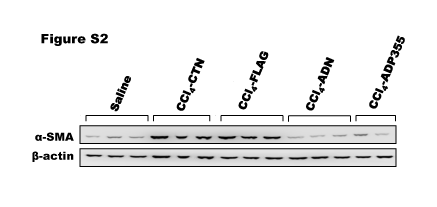

Supplement: Figure S2 — ADP355 attenuates CCl4-induced α-SMA expression. Representative Western blot for α-SMA liver lysates obtained from different cohorts (N = 3 mice/cohort). (TIF) [file pone.0110405.s002.tif]

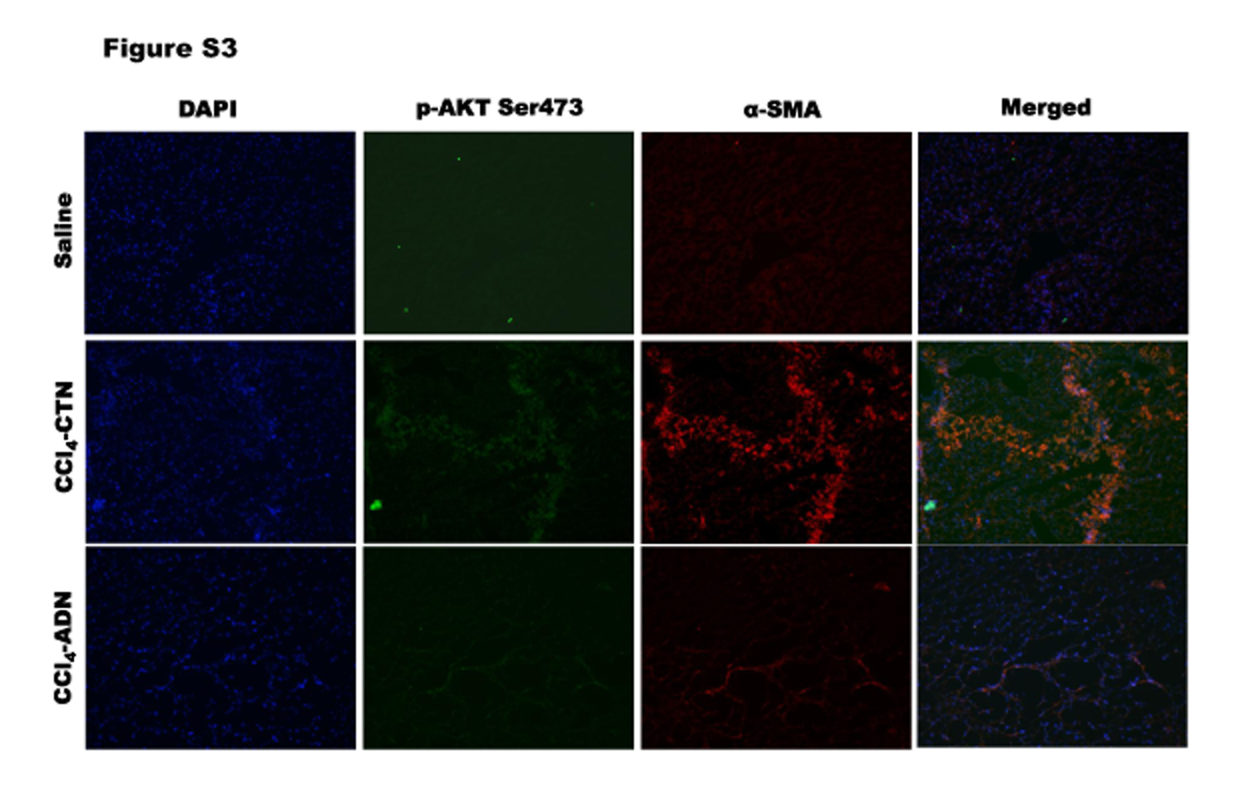

Supplement: Figure S3 — p-AKT co-localized with α-SMA positive cells: Representative images of immunofluorescent staining reveal both p-AKT and α-SMA co-localized in liver sections obtained from CCl4 gavaged mice. (TIF) [file pone.0110405.s003.tif]
